# Temporal Changes in the Genetic Diversity of *Plasmodium vivax* Merozoite Surface Protein-1 in Myanmar

**DOI:** 10.3390/pathogens10080916

**Published:** 2021-07-21

**Authors:** Haung Naw, Jung-Mi Kang, Mya Moe, Jinyoung Lee, Hương Giang Lê, Tuấn Cường Võ, Yi Yi Mya, Moe Kyaw Myint, Zaw Than Htun, Tong-Soo Kim, Ho-Joon Shin, Byoung-Kuk Na

**Affiliations:** 1 Department of Parasitology and Tropical Medicine, Institute of Health Sciences, College of Medicine, Gyeongsang National University, Jinju 52727, Korea; haungnaw23@gmail.com (H.N.); gjm9951001@hanmail.net (J.-M.K.); gianglee291994@gmail.com (H.G.L.); vtcuong241@gmail.com (T.C.V.); 2 Department of Convergence Medical Science, Gyeongsang National University, Jinju 52727, Korea; 3 Department of Medical Research Pyin Oo Lwin Branch, Pyin Oo Lwin 05062, Myanmar; myamoee.dmr@gmail.com (M.M.); yimya.dmrum@gmail.com (Y.Y.M.); dr.myintmoekyaw@gmail.com (M.K.M.); zawthanhtun@gmail.com (Z.T.H.); 4 Department of Tropical Medicine, Inha Research Institute for Medical Sciences, College of Medicine, Inha University, Incheon 22212, Korea; jylee5492@gmail.com (J.L.); tongsookim@inha.ac.kr (T.-S.K.); 5 Department of Microbiology, College of Medicine, Ajou University, Suwon 16499, Korea; hjshin@ajou.ac.kr; 6 Department of Biomedical Science, Graduate School of Ajou University, Suwon 16499, Korea

**Keywords:** *Plasmodium vivax*, merozoite surface protein-1, Myanmar, genetic diversity

## Abstract

Despite a significant decline in the incidence of malaria in Myanmar recently, malaria is still an important public health concern in the country. Although *Plasmodium falciparum* is associated with the highest incidence of malaria in Myanmar, the proportion of *P. vivax* cases has shown a gradual increase in recent years. The genetic diversity of *P. vivax* merozoite surface protein-1 block 5-6 (*pvmsp*-*1* ICB 5-6) in the *P. vivax* population of Myanmar was analyzed to obtain a comprehensive insight into its genetic heterogeneity and evolutionary history. High levels of genetic diversity of *pvmsp*-*1* ICB 5-6 were identified in the *P. vivax* isolates collected from Myanmar between 2013 and 2015. Thirty-nine distinct haplotypes of *pvmsp*-*1* ICB 5-6 (13 for Sal I type, 20 for recombinant type, and 6 for Belem type) were found at the amino acid level. Comparative analyses of the genetic diversity of *pvmsp*-*1* ICB 5-6 sequences in the recent (2013–2015) and the past (2004) *P. vivax* populations in Myanmar revealed genetic expansion of the *pvmsp*-*1* ICB 5-6 in recent years, albeit with a declined incidence. The recent increase in the genetic heterogeneity of Myanmar *pvmsp*-*1* ICB 5-6 is attributed to a combination of factors, including accumulated mutations and recombination. These results suggest that the size of the *P. vivax* population in Myanmar is sufficient to enable the generation and maintenance of genetic diversity, warranting continuous molecular surveillance of genetic variation in Myanmar *P. vivax*.

## 1. Introduction

Malaria is an important public health disease that is caused by *Plasmodium* species, which is transmitted by female *Anopheles* mosquito vectors. Although the global cases of malaria have declined substantially in recent decades, malaria is still a public health concern in many endemic countries. According to the World Health Organization, an estimated 229 million individuals worldwide were afflicted with malaria in 2019, including 409,000 deaths [1]. Myanmar is a country with malaria prevalence accounting for 31% of the reported cases in the Greater Mekong Subregion (GMS) [1]. A lack of effective vaccines and the emergence of drug resistance in malaria parasites hinder successful disease control in the country [2,3]. Four major species of *Plasmodium* infecting humans have been reported to be circulating in Myanmar [4]. Among them, the incidence of *Plasmodium falciparum* was the highest in the country in the last few decades, but the proportion of *P. vivax* infections has been gradually increasing in recent years [1,5,6].

Merozoite surface protein-1 of *P. vivax* (PvMSP-1) plays an important role in erythrocyte invasion and induces protective immunity in the host, and therefore is one of the foremost vaccine candidates [7,8,9]. PvMSP-1 is a 200-kDa protein encoded by *pvmsp-1*, which consists of seven conserved blocks flanked by six variables blocks, representing the sites with diverse insertions, deletions, recombination, and point mutations [10]. Due to these polymorphic patterns, *pvmsp-1* has been recognized as one of the reliable genetic markers for the analysis of *P. vivax* population dynamics. Despite PvMSP-1 being considered a plausible vaccine candidate, the genetic diversity of the antigen prevents immune recognition against *P. vivax* with different genotypes [11]. Genetic diversity hampers the development of a global vaccine against vivax malaria based on this antigen. Therefore, the monitoring of genetic diversity of *pvmsp-1* is necessary to elucidate the gene polymorphism and evolutionary aspects in the *P. vivax* population [11]. Previously, we identified and reported large genetic polymorphism involving *pvmsp-1* block 6 (ICB 5-6) in *P. vivax* isolates collected in Mandalay in Myanmar in 2004 [12]. The ICB 5-6 is a partial fragment of the block 6 variable region in the *pvmsp-1*, and shows diverse polymorphic characters caused by insertions, deletions, intra-allelic recombination, and point mutations [10,13,14]. The extreme genetic polymorphic nature of *pvmsp-1* ICB 5-6 has rendered it a reliable polymorphic marker for genetic structure analysis of *P. vivax* [10,13,14,15]. In the present study, we analyzed the genetic polymorphism of *pvmsp-1* ICB 5-6 in *P. vivax* isolates collected in the same areas of Myanmar between 2013 and 2015 to elucidate the time-series changes of the gene to evaluate its genetic heterogeneity and the evolutionary events in the *P. vivax* population of Myanmar.

## 2. Materials and Methods

### 2.1. Blood Sample Collection

**Figure 1:**
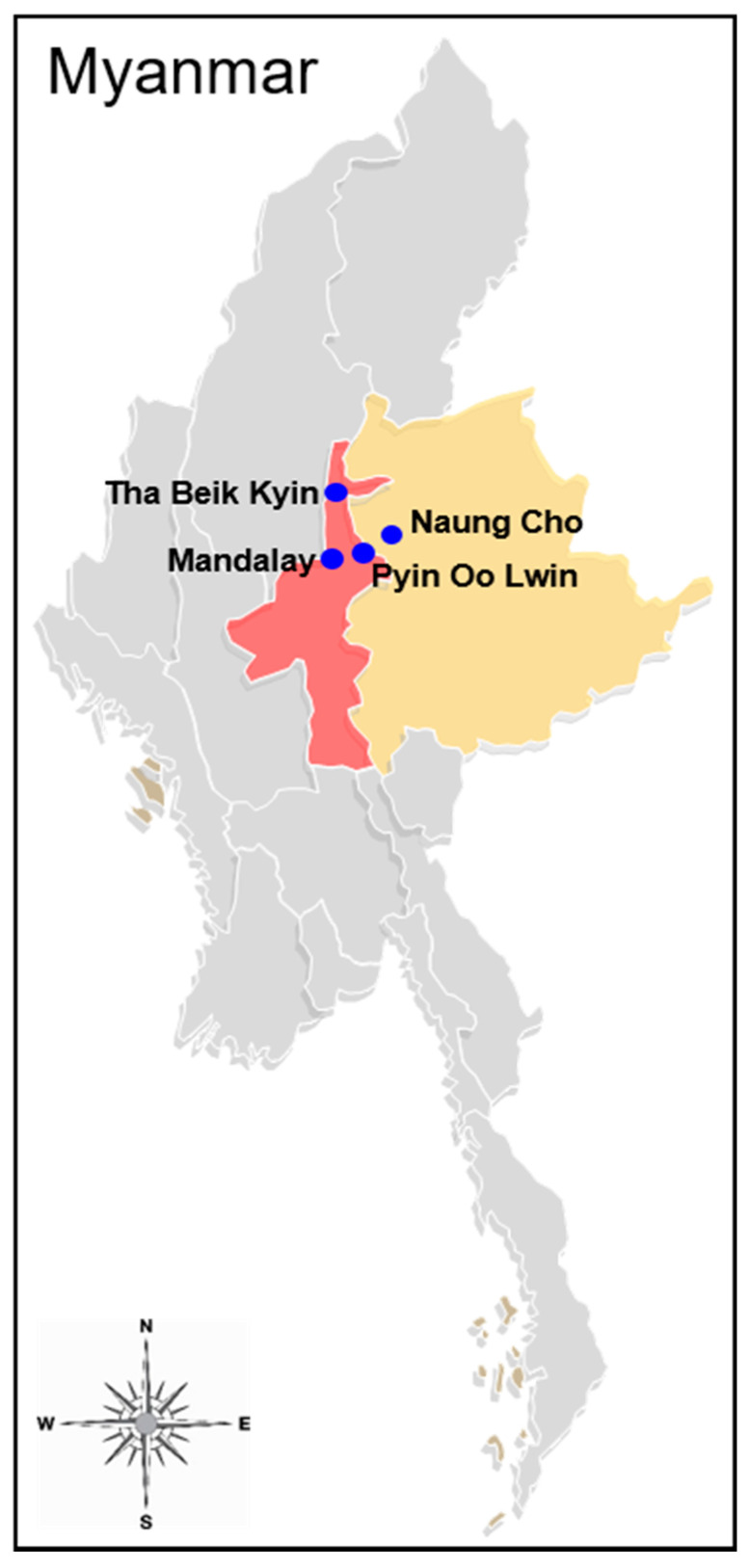
Map of Myanmar showing sample collection areas. Blue circles indicate blood sample collection sites. Yellow and red colors represent the Shan State and Mandalay Region, respectively.

Eighty-three blood samples used in this study were collected from *P. vivax*-infected symptomatic malaria patients in Naung Cho, Pyin Oo Lwin, Tha Beik Kyin, and Mandalay areas in Myanmar in community-based surveys from August 2013 to December 2015 (Figure 1). *P. vivax* infection was confirmed by Giemsa-stained thick and thin blood smear examination. All *P. vivax*-positive samples were further confirmed by a polymerase chain reaction (PCR) targeting the 18S ribosomal RNA (rRNA) gene [16]. Prior to drug treatment, the patients’ blood samples were collected on filter papers (Whatman 3 mm, GE Healthcare, Pittsburg, CA, USA), air-dried, and stored in sealed plastic bags at ambient temperature until use. Informed consent was obtained from all patients before blood collection. The study protocol was approved by the Ethics committee of the Ministry of Health, Myanmar (97/Ethics 2015), and the Biomedical Research Ethics Review Board of Inha University School of Medicine, Republic of Korea (INHA 15-013).

### 2.2. Genomic DNA Extraction

Genomic DNA was extracted from the dried blood spots using a QIAamp DNA Blood Kit (Qiagen, Hilden, Germany) [5] according to the manufacturer’s instructions.

### 2.3. Amplification and Sequence Analysis of pvmsp-1 ICB 5-6

The *pvmsp-1* ICB 5-6 region was amplified with nested-PCR using specific primer sets, as described previously [12]. Amplification was performed with thermal cycling conditions of initial denaturing at 94 °C for 10 min, 30 cycles of denaturation of 94 °C for 1 min, annealing at 52 °C for 1 min, and extension at 72 °C for 1 min, and the final extension at 72 °C for 10 min. Ex Taq DNA polymerase (Takara, Otsu, Japan) was used in all amplification steps to minimize possible amplification error. Each PCR product was analyzed on 1.5% agarose gel, purified from gel, and ligated into the T&A vector (Real Biotech Corporation, Banqiao City, Taiwan). Each ligation mixture was transformed into *Escherichia coli* DH5α competent cells (Real Biotech Corporation), and positive clones with appropriate insert were selected by colony PCR. The nucleotide sequences of the cloned gene were analyzed by automatic DNA sequencing with M13 forward and M13 reverse primers. Plasmids from at least two independent clones from each isolate were sequenced to verify sequence accuracy. Nucleotide and deduced amino acid sequences of *pvmsp-1* ICB 5-6 were analyzed using the EditSeq and Megalign programs in the DNASTAR package (DNASTAR, Madison, WI, USA). Nucleotide sequences of *pvmsp-1* ICB 5-6 obtained from the Myanmar *P. vivax* isolates were aligned with those corresponding to the Sal I (XM_001614792) and Belem (AF435594). The nucleotide sequences obtained in this study were deposited at GenBank under the accession numbers MW383137–MW383219 (Supplementary Table S1).

### 2.4. Temporal Change of Genetic Diversity in Myanmar pvmsp-1 ICB 5-6

To analyze temporal change of genetic diversity in Myanmar *pvmsp-1* during the last decade, the *pvmsp-1* ICB 5-6 sequences obtained in this study were compared with the 135 Myanmar *pvmsp-1* ICB 5-6 sequences (EU048257–EU048268), which were reported in the *P. vivax* samples collected in 2004 [12]. All sequences were comparatively analyzed with the Megalign program in the DNASTAR package (DNASTAR) and DnaSP ver. 5.10.00 [17] to determine subtype change, single nucleotide polymorphisms (SNPs), insertions, deletions, and recombination. The recombination parameter (R), which contained the effective population size and probability of recombination between adjacent nucleotides per generation, and the minimum number of recombination events (Rm) were measured using DnaSP ver. 5.10.00 [17]. Linkage disequilibrium (LD) between different polymorphic sites was computed in terms of the R^2^ index using DnaSP ver. 5.10.00. The R^2^ values were plotted against the nucleotide diversity distances with the two-tailed Fisher’s exact test of significance [17]. The phylogeny tree of Myanmar *pvmsp-1* ICB 5-6 was constructed with the neighbor-joining method using MEGA6 (http://www.megasoftware.net; accessed on 19 October 2020). The robustness of the nodes was assessed with 1000 replications. A haplotype network was constructed to investigate the relationships between the haplotypes using NETWORK software v.5.0 with the median-joining algorithm [18]. 

## 3. Results

### 3.1. Sequence Analysis of Myanmar pvmsp-1 ICB 5-6

**Figure 2:**
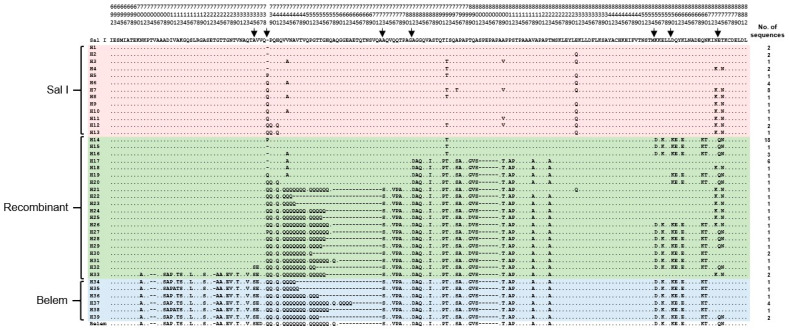
Genetic diversity of *pvmsp-1* ICB 5-6 in Myanmar *P. vivax* isolates. A total of 39 different haplotypes of Myanmar *pvmsp-1* ICB 5-6 were obtained. Multiple sequence alignment of deduced amino acid sequences of the 39 haplotypes were constructed corresponding to the amino acid sequences of the reference strains Sal I (XM_001614792) and Belem (AF435594). Amino acids identical to those of Sal I are represented by dots. The dashes represent gaps introduced to maximize the alignment. The predicted recombination sites are represented by arrows.

A total of 83 *pvmsp-1* ICB 5-6 sequences were successfully amplified in Myanmar *P. vivax* isolates collected between 2013 and 2015. Size polymorphisms of amino acid lengths ranging from 154 to 193 were identified. Based on sequence analysis, 39 distinct haplotypes (H1 to H39) were identified, which were grouped into three different allelic types, Sal I, Belem, and recombinant types (Figure 2). Recombinant types (H14 to H33) were the most prevalent (46/83, 55.4%), followed by Sal I (H1 to H13) (27/83, 32.5%) and Belem alleles (H34 to H39) (10/83, 12.1%). Sal I haplotypes showed multiple non-synonymous SNPs. H1 shared identical amino acid sequences with Sal I, but non-synonymous amino acid changes, including P739Q, H741Q, V744A, I792T, A795T, A809V, E831Q, N873K, and T875N, were detected in other haplotypes. Insertions of either Gln (Q) or Pro (P) between amino acid positions 738 and 739 (based on Sal I) were also identified in nine haplotypes (H5 to H13). Twenty haplotypes (H14 to H33) were classified into recombinant types involving predictive recombination events between Sal I and Belem types at seven positions (Figure 2). H14 was the most prevalent recombinant type, with a frequency of 21.7%. Heterogeneous Qs were found in the recombinant haplotypes. Six haplotypes (H14 to H19) did not harbor poly-Gln (poly-Q) repeats, but the other haplotypes showed different patterns of poly-Q repeats. Six haplotypes of Belem type (H34 to H39) also contained poly-Q repeats with different numbers of Q ranging from 10 to 27 for each haplotype (Figure 2). E874Q was identified in 10 recombinant and Belem haplotypes.

### 3.2. Temporal Changes in Myanmar pvmsp-1 ICB 5-6 in 2004 and in 2013–2015

**Figure 3:**
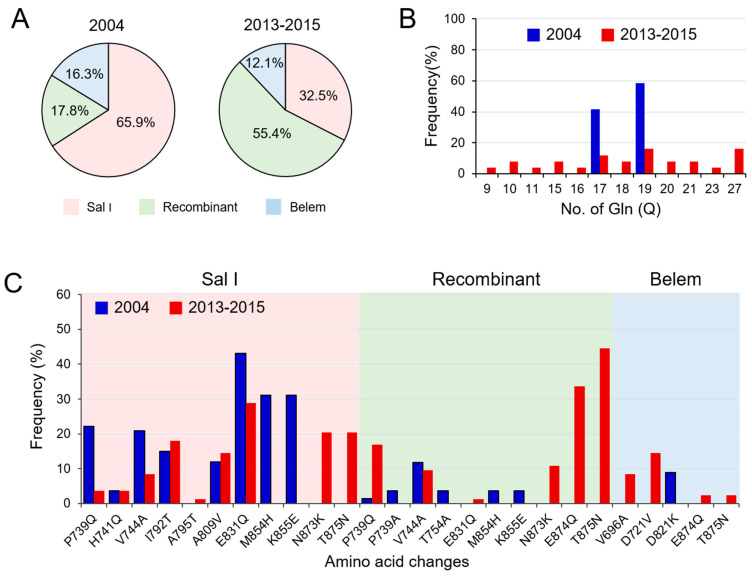
Temporal changes in the genetic diversity of Myanmar *pvmsp-1* ICB 5-6 in the previous year (2004) and recent years (2013–2015): (**A**) time-series change of allelic types. (**B**) Poly-Q repeats in Belem and recombinant types. (**C**) Amino acid changes. Amino acid changes based on Sal I (XM_001614792) are indicated.

**Figure 4:**
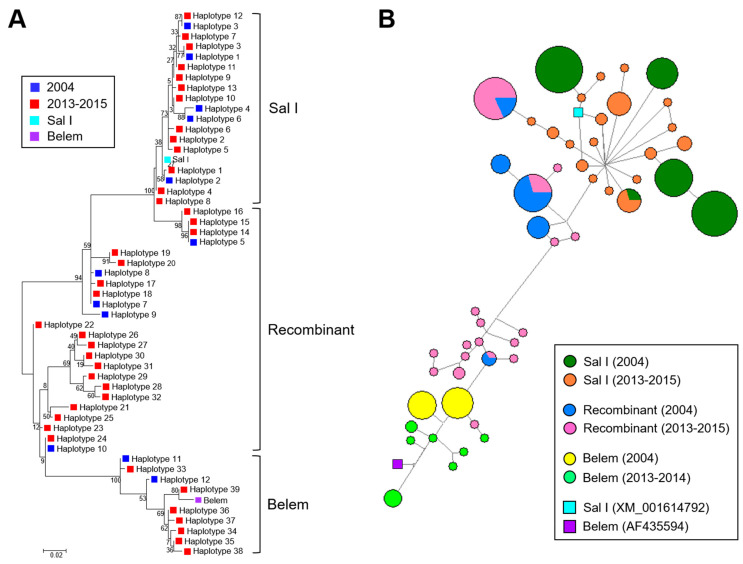
Phylogenetic analysis and haplotype network of Myanmar *pvmsp-1* ICB 5-6: (**A**) phylogenetic analysis. The phylogenetic tree was constructed with the neighbor-joining method using the MEGA6 program. Numbers on the branches indicate bootstrap proportions (1000 replicates). (**B**) Haplotype network. The haplotype network was constructed with 218 sequences from two study periods (2004 and 2013–2015) and 2 reference sequences, Sal I (XM_001614792) and Belem (AF435594), by using the median-joining algorithm implemented in network version 5.0.0.3 software. Pies represent the haplotypes and lines indicate connections between neighboring pies. The size of each pie implies frequency of the corresponding haplotype.

**Figure 5:**
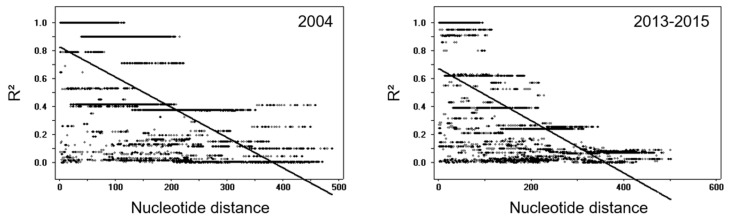
The linkage disequilibrium (LD) plot of Myanmar *pvmsp-1* ICB 5-6 between 2004 and 2013–2015. The LD plot showed non-random associations between nucleotide variants in *pvmsp-1* ICB 5-6 at different polymorphic sites. The R^2^ values were plotted against nucleotide distance using a two-tailed Fisher’s exact test for statistical significance.

Myanmar *pvmsp-1* ICB 5-6 showed different patterns of genetic heterogeneity between the previous sample year (2004) and recent years (2013–2015). A total of 12 different haplotypes (6 Sal I, 4 recombinant, and 2 Belem types) of *pvmsp-1* ICB 5-6 were previously identified in a 135 Myanmar *P. vivax* population reported in 2004 [12]. The overall genetic diversity of Myanmar *pvmsp-1* ICB 5-6 was greater in recent years than in the previous year. Although only 12 haplotypes were identified in 2004, 39 haplotypes were detected in recent years mainly due to amino acid polymorphisms, insertions, deletions, and recombination events. Haplotypes of Sal I type were prevalent in 2004 (65.9%), but recombinant haplotypes were most prevalent (55.4%) in 2013–2015 (Figure 3A). Poly-Q repeats in Belem and recombinant types were also more diverse in *pvmsp-1* ICB 5-6 identified in recent years. The number of Qs in haplotypes of recombinant and Belem types showed increased heterogeneity in recent years than in the previous year (Figure 3B). In 2004, only two forms of poly-Q repeats constructed with 17 or 19 Qs were identified. However, the number of Qs diversified in 2013–2015 and ranged from 9 to 27. Amino acid changes due to SNPs were also identified in Myanmar *pvmsp-1* ICB 5-6 from both the previous and recent sample years, but the amino acid positions and frequencies varied (Figure 3C). Three amino acid changes including A765T, N873K, and T875N were newly detected in Sal I types in recent years. Four amino acid changes were newly identified in the recombinant type (E831Q, N873K, E874Q, and T875N) and, similarly, four amino acid changes were also newly identified in the Belem type (V696A, D721V, E874Q, and T836N). Meanwhile, the amino acid changes identified in 2004, including M854H and K855E in Sal I types; P739A, T754A, M854H, and K855E in recombinant types; and D821K in Belem types, were not detected in the recent Myanmar *pvmsp-1* ICB 5-6. Eight amino acid changes (P739Q, H741Q, V744A, I792T, A809V, and E831Q in Sal I and P739Q and V744A in recombinant types) were generally found in *pvmsp-1* ICB 5-6 from both the previous and the recent years, albeit with varying frequencies. Phylogenetic analysis suggested the close genetic relatedness of *pvmsp-1* ICB 5-6 sequences between the parasites collected in 2004 and 2013–2015 (Figure 4A). Haplotype network analysis of Myanmar *pvmsp-1* ICB 5-6 revealed a total of 51 haplotypes in 218 Myanmar sequences (Figure 4B). As expected, the haplotype network showed two major clusters, Sal I and Belem. Sal I constituted the largest cluster, including 22 different branched haplotypes. Only one haplotype was shared by the *pvmsp-1* ICB 5-6 sequences identified in the previous year and in recent years. The other 21 haplotypes were individually occupied by sequences either from the previous year or the recent years. In the case of Belem types, haplotype diversity was greater in the *pvmsp-1* ICB 5-6 sequences from recent years than in those from the previous year. A total of seven distinct haplotypes were identified in the sequences from recent years, but only two haplotypes were from the previous year. Interestingly, diverse recombinant haplotypes were derived from Sal I and Belem. Seven recombinant haplotypes were branched from Sal I, including 2 haplotypes sharing sequences identified in the previous and recent years. Highly branched patterns of recombinant haplotypes originated from Belem. A total of 13 recombinant haplotypes were branched from Belem, including most of the haplotypes (12/13) identified in recent years, suggesting active genetic recombination in the Myanmar *pvmsp-1 *population. Indeed, Rm value has increased to seven in recent years compared to the previous year of three (Table 1). The LD index R^2^ plot for the Myanmar *pvmsp-1* ICB 5-6 in both the periods sharply declined across the analyzed regions, implying that intragenic recombination could be contributing to the genetic diversity of the Myanmar *pvmsp-1* populations (Figure 5). The reduction rate of LD index R^2^ was greater in Myanmar *pvmsp-1* ICB 5-6 in 2013–2015 compared to that in 2004.

## 4. Discussion

Myanmar aims to eliminate malaria by 2030. Progress has been made in reducing disease morbidity and mortality during the last decade, but malaria control in the country is still a challenge due to the heterogeneity of disease distribution and the emergence and spread of drug-resistant strains [19]. Understanding the genetic heterogeneity and evolutionary trend of malaria parasites in a malaria-endemic area or country is important to guide rational management strategies and provide fundamental insights into the design of an effective vaccine. 

In this study, time-series changes in genetic diversity and evolutionary trends of Myanmar *pvmsp-1 *ICB 5-6 were analyzed to obtain comprehensive insight into the genetic nature of the *P. vivax* population circulating in the Mandalay area, Myanmar. High genetic diversity of *pvmsp-1* ICB 5-6 was reported in the Myanmar *P. vivax* population in previous years [12]. Similarly, all three allelic types of *pvmsp-1* ICB 5-6, including Sal I, recombinant, and Belem types, were identified in the Myanmar *P. vivax* population analyzed in this study. However, the proportion of each allelic type differed between the previous year (2004) and recent years (2013–2015). The proportion of recombinant types was greatly increased in recent years (55.4%) compared to the previous sample year (17.8%). Meanwhile, the proportion of Sal I type decreased remarkably in recent years (32.5%) compared to the previous year (65.9%). 

Overall, the haplotype diversity of Myanmar *pvmsp-1* ICB 5-6 also increased in recent years compared with the previous year, although the incidence of malaria in the studied areas decreased remarkably in the last decade [16]. Twelve different haplotypes of *pvmsp-1* ICB 5-6 were previously identified from 135 Myanmar *P. vivax* isolates collected in 2004 [12]. However, 39 distinct haplotypes of *pvmsp-1* ICB 5-6 were detected in 83 samples in recent years (2013–2015). Haplotype network analysis of Myanmar *pvmsp-1* ICB 5-6 also suggests remarkable variation in haplotypes in recent years, with the appearance of new haplotypes and disappearance of pre-existing ones. The diversity of Sal I and Belem types of Myanmar *pvmsp-1* ICB 5-6 was driven primarily by SNPs. Only a single haplotype of Sal I type was shared by both sequences from the previous and recent years. Diverse Sal I and Belem haplotypes were detected recently. More remarkable haplotype changes, including highly branched patterns of recombinant haplotypes, were generated from Sal I and Belem in recent years. In particular, a total of 13 recombinant haplotypes were branched from Belem, including most of the newly generated haplotypes (12/13) in recent years. Active genetic recombination between Sal I and Belem types may be caused by either sexual recombination during meiosis or intrahelical strand slippage during DNA replication, resulting in novel recombinant haplotypes of Myanmar *pvmsp-1* ICB 5-6. The higher value of Rm and R^2^ observed in the recent *pvmsp-1* ICB 5-6 compared to those of the previous year support the theory that high levels of recombination events may have occurred in the gene in recent years, which render the genetic make-up of the gene more diverse. Insertions and deletions of Qs also affected the recent increase in heterogeneity of recombinant and Belem types. 

However, the factors contributing to the diversity of the Myanmar *pvmsp-1* population structure in the areas investigated in recent years compared with the previous year are unclear, despite the remarkable decline in the rate of malaria transmission in the last decade. Similar patterns of increased genetic diversity in recent years have been identified in *pfmsp-1* and *pfmsp-2* of the Myanmar *P. falciparum* population in the study area [20]. Such features are not clearly apparent in the *P. vivax* population in many other countries, even in low transmission areas and pre-elimination settings [5,21,22,23,24]. Relapses of distinct genotypes of *P. vivax* hypnozoites from prior infections accumulated in the liver may explain the increased genetic diversity despite the reduction in prevalence [21]. Asymptomatic carriers may also act as fundamental reservoirs for transmission and facilitate superinfection [25]. A substantial level of asymptomatic infections in the studied areas had been previously reported [5]. Active recombination events between different haplotypes or genotypes may also lead to the maintenance or generation of population genetic diversity due to the complexity of the genetic makeup [5,21,22,23,24]. As identified in this study, the high degree of genetic recombination between Sal I and Belem types of Myanmar *pvmsp-1*, which greatly increased the overall proportion of recombinant types, was expected in recent years. It may also be noteworthy to mention that the impact of migrants on the genetic diversity of the *P. vivax* population in the studied areas. Although malaria incidences have significantly declined in the central part of Myanmar in the last decade, migration from endemic areas or international border areas such as the Myanmar-China and Myanmar-Thailand borders has been increasing in recent years due to political instability and job recruiting. Large *P. vivax* reservoirs, including asymptomatic individuals, still maintain genetic diversity and transmission in the border areas with considerable spatial and temporal differentiations [6,26,27]. Further studies on the influx of *P. vivax* from other endemic areas or boarder areas and its contribution to the population diversity of *P. vivax* in Mandalay areas are also necessary to understand the causes of the increase in genetic diversity of the *P. vivax* population in the studied areas, and to provide guidelines for effective control measures.

Multiplicity of infection (MOI) is defined as the number of genetically different parasite genotypes co-infected in a single host, and it is an important indicator for measuring malaria transmission intensity [28]. Multi-clonal infections are commonly identified in malaria endemic areas, and these contribute to the genetic diversity of malaria parasites by rendering genetic recombination between different alleles [29,30,31]. Therefore, comparison of MOI between *P. vivax* isolates collected in 2004 and 2013–2015 would be informative when investigating the change in transmission intensity and its effect on the genetic diversity of the parasites. Unfortunately, direct comparison of MOI between the two parasite groups collected in 2004 and 2013–2015 was not possible in this study since the information for MOI in the parasites collected in 2004 was not available. In this study, we analyzed MOI of *P. vivax* isolates collected in 2013–2015 and found that the MOI was not high (around 1.0). Genotypes with low prevalence could not be successfully detected in our experiments, and the minor ones could be missed in the PCR and cloning processes. This procedure would be feasible when the *pvmsp-1* ICB 5-6 of minor alleles is a very similar size to those of the major alleles in a certain isolate. This highlights a technical limitation of our study and should be further investigated. Despite this, our results suggest an increased genetic diversity of *pvmsp-1* ICB 5-6 in the recent Myanmar *P. vivax* population compared to past ones.

Genetic diversity of *P. vivax* is one of the fundamental factors affecting transmission and immunity [11]. Genetic polymorphisms and haplotype diversity of vaccine candidate antigens could produce antigenic variations and enable parasites to escape host immune responses and abrogate immune recognition [10,11], which would hamper effective control and vaccine development. Continuous monitoring of the genetic diversity of vaccine candidate antigens including *pvmsp-1* is necessary to elucidate the genetic polymorphic nature and gene flow in the Myanmar *P. vivax* population.

## 5. Conclusions

Despite remarkable reductions in the incidence of malaria in the Mandalay area of Myanmar in the last decade, the *P. vivax* population in the area is increasing in genetic diversity, suggesting that the size of Myanmar *P. vivax* population is still sufficient to facilitate the generation and maintenance of genetic diversity. A combination of factors including accumulated mutations and recombination are the possible reasons for the recent increase in genetic diversity of *pvmsp-1* ICB 5-6 in the Myanmar *P. vivax* population. Given the limited number of *P. vivax* isolates analyzed over the years, a further analysis with larger number of Myanmar *P. vivax* isolates is warranted. Additional studies to determine the dynamics of genetic diversity and gene flow of other polymorphic markers may also be necessary to delineate the genetic structure and dynamics of *P. vivax* population in Myanmar.

## Figures and Tables

**Table 1:** Recombination events in Myanmar *pvmsp-1* ICB 5-6 between 2004 and 2013–2015.

Year	Ra	Rb	Rm
2004	0.000	0.001	3
2013–2015	0.058	3.2	7

Ra, recombination parameter between adjacent sites; Rb, recombination parameter for entire gene; Rm, minimum number of recombination events between adjacent sites.

## Data Availability

The data supporting the conclusions of this article are provided within the article. The original datasets analyzed in this study are available from the corresponding author upon request. The nucleotide sequences reported in this study have been deposited in the GenBank database under the accession numbers MW383137–MW383219.

## Supplementary Materials

The following are available online at https://www.mdpi.com/article/10.3390/pathogens10080916/s1, Table S1: Summary of Myanmar *pvmsp-1* ICB 5-6 haplotype information.

## References

[1] World Health Organization (2020). World Malaria Report 2020: 20 Years of Global Progress and Challenges.

[2] World Health Organization (2017). World Malaria Report 2016.

[3] Mu T.T., Sein A.A., Kyi T.T., Min M., Aung N.M., Anstey N.M., Kyaw M.P., Soe C., Kyi M.M., Hanson J. (2016). Malaria incidence in Myanmar 2005–2014: Steady but fragile progress towards elimination. Malar. J..

[4] Lin K., Kano S., Tongol-Rivera P. (2005). Malaria Control in Myanmar.

[5] Kang J.M., Cho P.Y., Moe M., Lee J., Jun H., Lee H.W., Ahn S.K., Kim T.I., Pak J.H., Myint M.K. (2017). Comparison of the diagnostic performance of microscopic examination with nested polymerase chain reaction for optimum malaria diagnosis in Upper Myanmar. Malar. J..

[6] Huang F., Zhang L., Xue J.B., Zhou H.N., Thi A., Zhang J., Zhou S.S., Xia Z.G., Zhou X.N. (2020). From control to elimination: A spatial-temporal analysis of malaria along the China-Myanmar border. Infect. Dis. Poverty.

[7] Valderrama-Aguirre A., Quintero G., Gómez A., Castellanos A., Pérez Y., Méndez F., Arévalo-Herrera M., Herrera S. (2005). Antigenicity, immunogenicity, and protective efficacy of *Plasmodium vivax* MSP1 Pv200L: A potential malaria vaccine subunit. Am. J. Trop. Med. Hyg..

[8] Herrera S., Corradin G., Arévalo-Herrera M. (2007). An update on the search for a *Plasmodium vivax* vaccine. Trends Parasitol..

[9] Versiani F.G., Almeida M.E., Mariuba L.A., Orlandi P.P., Nogueira P.A. (2013). *N*-terminal *Plasmodium vivax* merozoite surface protein-1, a potential subunit for malaria vivax vaccine. Clin. Dev. Immunol..

[10] Putaporntip C., Jongwutiwes S., Sakihama N., Ferreira M.U., Kho W.G., Kaneko A., Kanbara H., Hattori T., Tanabe K. (2002). Mosaic organization and heterogeneity in frequency of allelic recombination of the *Plasmodium vivax* merozoite surface protein-1 locus. Proc. Natl. Acad. Sci. USA.

[11] Arnott A., Barry A.E., Reeder J.C. (2012). Understanding the population genetics of *Plasmodium vivax* is essential for malaria control and elimination. Malar. J..

[12] Moon S.U., Lee H.W., Kim J.Y., Na B.K., Cho S.H., Lin K., Sohn W.M., Kim T.S. (2009). High frequency of genetic diversity of *Plasmodium vivax* field isolates in Myanmar. Acta Trop..

[13] Del Portillo H.A., Longacre S., Khouri E., David P.H. (1991). Primary structure of the merozoite surface antigen 1 of *Plasmodium vivax* reveals sequences conserved between different Plasmodium species. Proc. Natl. Acad. Sci. USA.

[14] Ruan W., Zhang L.L., Feng Y., Zhang X., Chen H.L., Lu Q.Y., Yao L.N., Hu W. (2017). Genetic diversity of *Plasmodium vivax* revealed by the merozoite surface protein-1 icb5-6 fragment. Infect. Dis. Poverty.

[15] Craig A.A., Kain K.C. (1996). Molecular analysis of strains of *Plasmodium vivax* from paired primary and relapse infections. J. Infect. Dis..

[16] Snounou G., Viriyakosol S., Jarra W., Thaithong S., Brown K.N. (1993). Identification of the four human malaria parasite species in field samples by the polymerase chain reaction and detection of a high prevalence of mixed infections. Mol. Biochem. Parasitol..

[17] Librado P., Rozas J. (2009). DnaSP v5: A software for comprehensive analysis of DNA polymorphism data. Bioinformatics.

[18] Bandelt H.J., Forster P., Röhl A. (1999). Median-joining networks for inferring intraspecific phylogenies. Mol. Biol. Evol..

[19] (2016). National Strategic Plan: Intensifying Malaria Control and Accelerating Progress towards Malaria Elimination (2016–2020).

[20] Lê H.G., Kang J.M., Jun H., Lee J., Thái T.L., Myint M.K., Aye K.S., Sohn W.M., Shin H.J., Kim T.S. (2019). Changing pattern of the genetic diversities of *Plasmodium falciparum* merozoite surface protein-1 and merozoite surface protein-2 in Myanmar isolates. Malar. J..

[21] Koepfli C., Ross A., Kiniboro B., Smith T.A., Zimmerman P.A., Siba P., Mueller I., Felger I. (2011). Multiplicity and diversity of *Plasmodium vivax* infections in a highly endemic region in papua New Guinea. PLoS Negl. Trop. Dis..

[22] Gunawardena S., Ferreira M.U., Kapilananda G.M.G., Wirth D.F., Karunaweera N.D. (2014). The Sri Lankan paradox: High genetic diversity in *Plasmodium vivax* populations despite decreasing levels of malaria transmission. Parasitology.

[23] Li Y.C., Wang G.Z., Meng F., Zeng W., He C.H., Hu X.M., Wang S.Q. (2015). Genetic diversity of *Plasmodium vivax* population before elimination of malaria in Hainan Province, China. Malar. J..

[24] Kang J.M., Lee J., Cho P.Y., Kim T.I., Sohn W.M., Park J.W., Kim T.S., Na B.K. (2016). Dynamic changes of *Plasmodium vivax* population structure in South Korea. Infect. Genet. Evol..

[25] Vallejo A.F., Chaparro P.E., Benavides Y., Álvarez Á., Quintero J.P., Padilla J., Arévalo-Herrera M., Herrera S. (2015). High prevalence of sub-microscopic infections in Colombia. Malar. J..

[26] Tipmontree R., Fungladda W., Kaewkungwal J., Tempongko M.A., Schelp F.P. (2009). Migrants and malaria risk factors: A study of the Thai-Myanmar border. Southeast Asian J. Trop. Med. Public Health.

[27] Li Y., Hu Y., Zhao Y., Wang Q., Ngassa Mbenda H.G., Kittichai V., Lawpoolsri S., Sattabongkot J., Menezes L., Liu X. (2020). Dynamics of *Plasmodium vivax* populations in border areas of the Greater Mekong sub-region during malaria elimination. Malar. J..

[28] Kolakovich K.A., Ssengoba A., Wojcik K., Tsuboi T., Al-Yaman F., Alpers M., Adams J.H. (1996). *Plasmodium vivax*: Favored gene frequen cies of the merozoite surface protein-1 and the multiplicity of infection in a malaria endemic region. Exp. Parasitol..

[29] Fola A.A., Harrison G.L.A., Hazairin M.H., Barnadas C., Hetzel M.W., Iga J., Siba P.M., Mueller I., Barry A.E. (2017). Higher complexity of infection and genetic diversity of *Plasmodium vivax* than *Plasmodium falciparum* across all malaria transmission zones of Papua New Guinea. Am. J. Trop. Med. Hyg..

[30] Zhang C.L., Zhou H.N., Liu Q., Yang Y.M. (2019). Genetic polymorphism of merozoite surface proteins 1 and 2 of *Plasmodium falciparum* in the China-Myanmar border region. Malar. J..

[31] Võ T.C., Lê H.G., Kang J.M., Naw H., Fan C.K., Trinh N.T.M., Quang H.H., Na B.K. (2021). Molecular surveillance of malaria in the Central Highlands, Vietnam. Parasitol. Int..

