# Supplementary material for: Temporal Changes in the Genetic Diversity of Plasmodium vivax Merozoite Surface Protein-1 in Myanmar
Source: Pathogens. 2021 Jul 21;10(8):916. doi: 10.3390/pathogens10080916 (PMC8398579; doi:10.3390/pathogens10080916)
Supplement: Supplementary file 1 [file pathogens-10-00916-s001.zip › pathogens-1233415-SI.pdf]

# Supplementary

**Table S1.** Summary of Myanmar *pvmsp-1* ICB 5-6 haplotype information.

| Year | Haplotype | Number of sequences in Myanmar *P. vivax* isolates | Genbank accession numbers |
|---|---|---|---|
| 2004 | 1 | 12 | EU048257 |
| | 2 | 26 | EU048258 |
| | 3 | 5 | EU048259 |
| | 4 | 25 | EU048260 |
| | 5 | 4 | EU048261 |
| | 6 | 17 | EU048262 |
| | 7 | 11 | EU048263 |
| | 8 | 6 | EU048264 |
| | 9 | 5 | EU048265 |
| | 10 | 2 | EU048266 |
| | 11 | 12 | EU048267 |
| | 12 | 10 | EU048268 |
| 2013-2015 | 1 | 2 | MW383145, MW383146 |
| | 2 | 2 | MW383143, MW383176 |
| | 3 | 1 | MW383144 |
| | 4 | 2 | MW383137, MW383139 |
| | 5 | 1 | MW383177 |
| | 6 | 4 | MW383153, MW383165, MW383166, MW383178 |
| | 7 | 8 | MW383149, MW383150, MW383151, MW383155, MW383163, MW383179, MW383181, MW383182 |
| | 8 | 1 | MW383162 |
| | 9 | 1 | MW383164 |
| | 10 | 1 | MW383167 |
| | 11 | 1 | MW383180 |
| | 12 | 2 | MW383184, MW383185 |
| | 13 | 1 | MW383183 |
| | 14 | 18 | MW383147, MW383148, MW383152, MW383154, MW383156, MW383157, MW383158, MW383159, MW383160, MW383161, MW383168, MW383169, |

| | | | |
|---|---|---|---|
| | | | MW383170, MW383171, MW383172, MW383173, MW383174, MW383175 |
| | 15 | 1 | MW383140 |
| | 16 | 3 | MW383138, MW383141, MW383142 |
| | 17 | 6 | MW383187, MW383188, MW383189, MW383190, MW383191, MW383192 |
| | 18 | 1 | MW383186 |
| | 19 | 1 | MW383193 |
| | 20 | 1 | MW383194 |
| | 21 | 1 | MW383195 |
| | 22 | 1 | MW383197 |
| | 23 | 1 | MW383198 |
| | 24 | 1 | MW383199 |
| | 25 | 1 | MW383201 |
| | 26 | 1 | MW383205 |
| | 27 | 1 | MW383196 |
| | 28 | 1 | MW383200 |
| | 29 | 1 | MW383204 |
| | 30 | 2 | MW383202, MW383203 |
| | 31 | 1 | MW383206 |
| | 32 | 1 | MW383207 |
| | 33 | 2 | MW383208, MW383211 |
| | 34 | 1 | MW383212 |
| | 35 | 1 | MW383213 |
| | 36 | 1 | MW383214 |
| | 37 | 4 | MW383215, MW383216, MW383217, MW383218 |
| | 38 | 1 | WM383210 |
| | 39 | 2 | MW383209, MW383219 |

Each haplotype was defined based on sequence and size variations.
